# Lipid signaling: facets of a versatile cell communication strategy in health and disease

**DOI:** 10.1007/s00424-024-03034-8

**Published:** 2024-11-11

**Authors:** Erich Gulbins, Josef Pfeilschifter

**Affiliations:** 1 Institut Für Molekularbiologie, Universitätsklinikum Essen, Universität Duisburg-Essen, Essen, Germany; 2 Institut für Allgemeine Pharmakologie und Toxikologie, Goethe-Universität, Frankfurt am Main, Germany

## Main text

Sphingolipids are a class of lipids whose outstanding biochemical and biomedical significance has emerged during the past 30 years. The targeting of sphingolipids is presently being developed as a treatment for serious human diseases such as cancer, bacterial and viral infections, inflammation, and neurodegenerative and neuropsychiatric disorders. Ceramides are a subfamily of sphingolipids in which the amino alcohol sphingosine is linked to a fatty acid. The esterification of ceramides with phosphorylcholine results in the formation of sphingomyelin. The hydrolysis of sphingomyelin into ceramide is catalyzed by sphingomyelinases, whereas the hydrolysis of ceramide into sphingosine is mediated by ceramidases. Sphingosine is then phosphorylated to sphingosine-1-phosphate.

It is now been firmly established that sphingolipids, in particular ceramide, sphingosine, and sphingosine-1-phosphate, are essential regulators of many fundamental cellular processes, such as cell death, but also differentiation and proliferation, receptor signaling, adhesion, embryogenesis, cancer, and immune regulation. The explosion of our knowledge about sphingolipids was facilitated by biochemical and biophysical studies of the signaling and cellular properties of sphingolipids, in particular ceramide, sphingosine, and sphingosine-1-phosphate; the cloning of enzymes involved in sphingolipid metabolism; the development of genetic models for determining the physiological role of sphingolipids; and the characterization of biochemical, biophysical, and optical methods of detecting and measuring the levels of sphingolipids. These insights into the biochemistry and cell biology of sphingolipids fundamentally changed our understanding of the ways in which cells are organized and function and how these lipids determine the pathogenesis and pathophysiology of important diseases. It is now well established that ceramide, sphingosine, and sphingosine-1-phosphate perform fundamental pathophysiological functions in malignant tumors, bacterial and viral infectious diseases, cystic fibrosis, inflammation, acute lung failure, sepsis, neuropsychiatric disorders such as major depressive disorder, atherosclerosis, storage diseases, neuroinflammatory diseases such as multiple sclerosis, and liver and renal pathologies.

The clinical significance of sphingolipids is exemplified by the finding that fingolimod, a blocker of sphingosine-1-phosphate receptors, has been developed to treat severe multiple sclerosis. Most antidepressants act by blocking acid sphingomyelinase, which converts sphingomyelin to ceramide. Several drugs targeting ceramidases, sphingomyelinases, and sphingosine-1-phosphate receptors are in development for the treatment of cancer, immune disorders, bacterial and viral infections, and major depressive disorder; these drugs are very exciting novel options for treating these severe and very common human diseases.

These examples underline the great potential of novel therapies based on a targeted modification of sphingolipids. The field of “Biomedicine of Sphingolipids” is currently in a very exciting transition from basic science to clinical application.

The present special issue reflects the multiple biological functions of sphingolipids and addresses many of the questions outlined above. Current developments in this innovative research area were presented and discussed at the Leopoldina Symposium “Lipid Signalling 2023” in Frankfurt am Main, organized by Erich Gulbins ML (Essen) and Josef Pfeilschifter ML (Frankfurt am Main) and supported by the German Research Organization (SFB 1039). It deals with evolutionary questions on how the pathways of sphingolipid metabolism developed [2], on the role of sphingosine 1-phosphate and sphingosine kinases in cellular Ca^2+^ homeostasis [11], the regulation of autophagy by sphingosine-1-phosphate and derivatives [9], and the role of sphingosine-1-phosphate in different biological systems such as the response of the central nervous system to stress [1, 3, 4] and its role in the development of chronic renal disease [3, 10]. The special issue also discusses novel findings on biological functions of ceramide in tumorigenesis [6] and the interaction of ceramide with phosphatidic acid in major depression [9]. Cancer-related questions are also investigated in an article dealing how fatty acids regulate immune checkpoints on cancer cells [5]. The special issue also broadens the view to fatty acid-derived mediators, in particular leukotrienes. Specifically, the special issue characterizes lipoxygenase isoforms and describes the discovery of novel 5-lipoxygenase inhibitors [7, 8].

With deepest sadness, we had to accept that our esteemed colleague Andrea Huwiler passed away on Monday, December 18, 2023. Her unexpected departure has left us all in shock and mourning. Andrea Huwiler was supposed to act as guest editor of this special issue of Pflügers Archive together with us. Therefore, this special issue is dedicated to our friend and colleague, Andrea Huwiler. We gratefully acknowledge the lively and ingenious scientific discussions we shared with her during many years.

## Data Availability

No datasets were generated or analysed during the current study.

## References

[1] Fischer C, Thomas D, Gurke R, Tegeder I (2024) Brain region specific regulation of anandamide (down) and sphingosine-1- phosphate (up) in association with anxiety (AEA) and resilience (S1P) in a mouse model of chronic unpredictable mild stress. Pflügers Arch - Eur J Physiol Collect: Special Issue: Signalling by Fatty Acid Derivatives and Sphingolipids in Health and Disease 10.1007/s00424-024-03012-0 PMC11582197 39177699

[2] Futerman AH (2024) Why do we study sphingolipids? Pflügers Arch - Eur J Physiol Collect: Special Issue: Signalling by Fatty Acid Derivatives and Sphingolipids in Health and Disease

[3] Glueck M, Lucaciu A, Subburayalu J, Kestner RI, Pfeilschifter W, Vutukuri R, Pfeilschifter J (2024) Atypical sphingosine-1-phosphate metabolites – biological implications of alkyl chain length. Pflügers Arch - Eur J Physiol Collect: Special Issue: Signalling by Fatty Acid Derivatives and Sphingolipids in Health and Disease 10.1007/s00424-024-03018-8 PMC11582160 39297971

[4] Kuo A, Hla T (2024) Regulation of cellular and systemic sphingolipid homeostasis. Nature Rev Mol Cell Biol 25:802–821 38890457 10.1038/s41580-024-00742-y PMC12034107

[5] Kur IM, Weigert A (2024) Phosphatidylserine externalization as immune checkpoint in cancer. Pflügers Arch - Eur J Physiol Collect: Special Issue: Signalling by Fatty Acid Derivatives and Sphingolipids in Health and Disease 10.1007/s00424-024-02948-7 PMC11582130 38573347

[6] Merz N, Hartel JC, Grösch S (2024) How ceramides affect the development of colon cancer: from normal colon to carcinoma. Pflügers Arch - Eur J Physiol Collect: Special Issue: Signalling by Fatty Acid Derivatives and Sphingolipids in Health and Disease 10.1007/s00424-024-02960-x PMC11582153 38635059

[7] Molitor M, Menge A, Mandel S, George S, Müller S, Knapp S, Hofmann B, Steinhilber D, Häfner AK (2024) Unlocking the potential: unveiling tyrphostins with Michael-reactive cyanoacrylate motif as promising inhibitors of human 5-lipoxygenase. Pflügers Arch - Eur J Physiol Collect: Special Issue: Signalling by Fatty Acid Derivatives and Sphingolipids in Health and Disease 10.1007/s00424-024-03019-7 PMC11582101 39347835

[8] Palmer MA, Benatzy Y, Brüne B (2024) Murine Alox8 versus the human ALOX15B ortholog: differences and similarities. Pflügers Arch - Eur J Physiol Collect: Special Issue: Signalling by Fatty Acid Derivatives and Sphingolipids in Health and Disease 10.1007/s00424-024-02961-w PMC11582214 38637408

[9] Schiller M, Wilson GC, Keitsch S, Soddemann M, Wilker B, Edwards MJ, Scherbaum N, Gulbins E (2024) Phosphatidic acid is involved in regulation of autophagy in neurons in vitro and in vivo. Pflügers Arch - Eur J Physiol Collect: Special Issue: Signalling by Fatty Acid Derivatives and Sphingolipids in Health and Disease 10.1007/s00424-024-03026-8 PMC11582205 39375214

[10] Schwalm S, Manaila R, Oftring A, Schaefer L, von Gunten S, Pfeilschifter J (2024) The contribution of the sphingosine 1-phosphate signaling pathway to chronic kidney diseases: recent findings and new perspectives. Pflügers Arch - Eur J Physiol Collect: Special Issue: Signalling by Fatty Acid Derivatives and Sphingolipids in Health and Disease 10.1007/s00424-024-03029-5 PMC11582123 39384640

[11] Volk LM, Bruun JE, Trautmann S, Thomas D, Schwalm S, Pfeilschifter J, Meyer zu Heringdorf D (2024) A role for plasma membrane Ca^2+^ ATPases in regulation of cellular Ca^2+^ homeostasis by sphingosine kinase-1. Pflügers Arch - Eur J Physiol Collect: Special Issue: Signalling by Fatty Acid Derivatives and Sphingolipids in Health and Disease 10.1007/s00424-024-03027-7 PMC11582158 39392480

